# Sex-Based Differences in Outcomes for Glioblastoma Patients Treated with Hypofractionated Chemoradiotherapy

**DOI:** 10.3390/cancers17213486

**Published:** 2025-10-30

**Authors:** Oscar Padilla, Masih Tazhibi, Nicholas McQuillan, Elizabeth J. Buss, Michael B. Sisti, Jeffrey N. Bruce, Guy M. McKhann, Simon K. Cheng, Tony J. C. Wang

**Affiliations:** 1 Department of Radiation Oncology, Columbia University Irving Medical Center, New York, NY 10032, USA; 2 Department of Radiation Oncology, Icahn School of Medicine at Mount Sinai, New York, NY 10029, USA; 3 Harvard Medical School, Boston, MA 02115, USA; 4 Department of Neurological Surgery, Columbia University Irving Medical Center, New York, NY 10029, USA

**Keywords:** glioblastoma, sex differences, survival, elderly, low performance status, demographics

## Abstract

**Simple Summary:**

Glioblastoma is a fatal brain tumor treated with surgery and chemoradiotherapy. In addition to known clinical and tumor related factors, certain demographic factors like sex, insurance status and income level have been recently reported to prognosticate for outcomes in patients receiving standard long-course chemoradiotherapy. However, it is not known whether these demographic factors prognosticate in patients who receive short-course chemoradiotherapy, an abbreviated treatment alternative reserved for elderly or low functional status patients. Identifying prognostic factors is important for refining personalized management approaches for this critically ill population. In this study, we investigate the role of demographic factors in patients receiving short-course chemoradiotherapy and find that female sex predicts for shorter time to tumor recurrence and death. These findings are first-in-kind and are contrary to what has been reported in patients who receive long-course chemoradiotherapy, highlighting the need to further investigate the impact of sex on response to different treatment regimens in glioblastoma.

**Abstract:**

Background/Objective: Elucidation of prognostic factors is key to personalizing management approach for patients with glioblastoma (GBM). In patients who are treated with conventionally fractionated radiotherapy (cvRT), sex and other demographic variables (e.g., income level) were recently found to predict for treatment outcomes. However, it is unknown whether these factors predict for outcomes in elderly or poor performance status patients who receive hypofractionated RT (hyRT). In this study, we assess the association of clinical and non-clinical factors to outcomes in GBM patients treated with hyRT concurrent with temozolomide (TMZ). Methods: The records of 61 adult patients with newly diagnosed GBM consecutively treated at our institution with post-operative hyRT (4005 cGy in 15 daily fractions) and TMZ were retrospectively analyzed. Established clinical variables as well as key demographic variables were compared using chi-squared tests. Kaplan–Meier analyses were used to compare overall survival (OS) and progression-free survival (PFS) between clinical and demographic subgroups. Multivariate modeling was performed using Cox proportional hazards regression. Results: Female and male patients composed 44.3% and 55.7% of the study population, respectively, and did not differ significantly in their clinical or tumor characteristics. Most patients were 65 years or older (85.2%), and over half resided in middle/high-income regions (55.7%) and were privately insured (55.7%). On an univariate analysis, female sex was associated with shorter OS (median 10.0 months vs. 13.3 months in males, *p* = 0.0224) and PFS (median 3.00 months vs. 4.60 months in males, *p* = 0.0134). Female sex remained significantly associated with inferior outcomes on multivariate analysis. Income level, type of insurance and marital status were not significantly associated with treatment outcomes. Conclusion: Our study is the first to report sex differences in GBM outcomes following hyRT-TMZ. Contrary to responses following cvRT-TMZ, females appear to have inferior outcomes after hyRT-TMZ versus males. Further investigation is warranted to define the optimal treatment approach for sex subgroups in GBM.

## 1. Introduction

Glioblastoma (GBM) is the most common primary malignant brain tumor in adults, occurring at an annual age-adjusted incidence rate of 3.19/100,000, and bearing a dismal prognosis of 12 to 15 months from diagnosis [1]. Population studies have shown that survival in GBM patients declines with increasing age and lower performance status, with an approximate median survival of six to nine months [2,3]. Management of GBM in this poor prognosis cohort is difficult given the higher incidence of comorbidities and increased risk of side-effects [4]. However, prior phase 3 studies demonstrate survival gains with the use of hypofractionated radiotherapy (hyRT) and concurrent temozolomide (TMZ) in this older, frail population [5,6,7].

While age and performance status, in addition to extent of resection (EOR) and methylguanine-DNA methyltransferase (MGMT) promoter methylation status, are well-established prognostic factors, sex and other demographic factors have been recently shown to predict for outcomes in GBM [8,9,10,11,12]. In a recent National Cancer Database (NCDB) analysis, investigators identified male sex, government insurance status and low-income as factors associated with diminished overall survival (OS) in GBM [11]. Moreover, the NRG Radiation Therapy Oncology Group (RTOG), in their 2021 GBM nomogram, identified sex as a significant prognostic factor for OS [13]. These results suggest that certain GBM demographics may be particularly vulnerable to worsened outcomes. However, these prior studies focused exclusively on patients treated with conventionally fractionated RT (cvRT) and TMZ [14,15]. As such, the prognostic impact of sex and other demographic factors remains unknown in GBM patients treated with hyRT-TMZ.

Delineation of potential prognostic factors, clinical and demographic alike, is important in personalizing the management approach for GBM patients. This is particularly critical for patients receiving hyRT, in whom non-clinical prognostic factors are less understood and whose survival is shorter than their counterparts receiving cvRT. In the present study, we conducted a retrospective analysis to determine if sex and demographic factors affect disease outcomes in a cohort of GBM patients treated with hyRT and TMZ. We find that female sex is associated with worse OS and progression-free survival (PFS), contrary to what is observed in patients treated with cvRT-TMZ [16,17].

## 2. Methods

### 2.1. Study Cohort

Between 2013 and 2019, 61 adult patients (age ≥ 18 years) with newly diagnosed, pathology-proven GBM completed curative intent hyRT-TMZ at our institution. Patients were consecutively treated by a multidisciplinary team of neurosurgeons, radiation oncologists and neuro-oncologists. Patients were excluded if they had received previous brain irradiation, did not complete the full course of radiotherapy, were treated with a non-hyRT schedule and/or did not receive concurrent TMZ. Patients were followed up on until time of death, which occurred in 59 of 61 patients.

### 2.2. Treatment

All 61 patients underwent surgery in the form of craniotomy and maximal feasible resection. Extent of resection (EOR) was determined by post-operative brain magnetic resonance imaging (MRI). All patients completed adjuvant of external-beam radiotherapy to the post-operative bed and T1- and fluid attenuated inversion recovery (FLAIR)-enhancing regions on post-operative MRI, to a total dose of 4005 cGy in 15 daily fractions as part of the curative intent hypofractionated protocol at our institution. TMZ was administered concurrently with hyRT in all patients.

### 2.3. Statistical Analyses

Overall survival (OS) time was measured from the date of surgery to the date of death or last follow-up for living patients. Progression-free survival (PFS) time was calculated from the end of radiotherapy to the date of recurrence, which was determined by a follow-up MRI and/or surgical pathology. The following variables were assessed: age, sex, Karnofsky Performance Scale (KPS) score, race/ethnicity, insurance type, marital status, income based on zip code as reported by the U.S. Census Bureau [18], EOR, MGMT promoter methylation status and Charlson Comorbidity Index score. For zip code income, Zip Code Tabulation Areas data were obtained from the U.S. Census Bureau and cross-referenced with the poverty threshold for our region in a given year; zip codes with a median income below this threshold were categorized as ‘low’ and those above the threshold as ‘middle/high.’ Comparisons between variables were performed using the Chi-squared test. The Kaplan–Meier method was used to calculate the probability of tumor control and survival; 95% confidence intervals were estimated according to Altman’s method [19]. OS and PFS curves were compared in a univariate analysis using the log-rank test. Hazard ratios (HR) with confidence intervals (CI) were calculated using the method described by Klein and Moeschberger [20]. For continuous variables such as age, univariate Cox proportional hazards regression was used to determine association with OS and PFS [21]. Variables that were found to be significant (*p*-value < 0.05) were considered in the multivariate analysis. Multivariate analysis was performed by Cox proportional hazards regression [21].

## 3. Results

### 3.1. Composite Demographics

A total of 61 patients who met inclusion and exclusion criteria were identified for our study. Baseline patient demographics and treatment factors are shown in Table 1. Thirty-four patients were male and twenty-seven were female. The median age of the entire cohort was 71.0 years (interquartile range (IQR) 9.5) and 70.5 years (IQR 10) for males, and 72.0 years (IQR 11) for females. The majority of patients (85.2%) were 65 years or older and considered elderly as per historical GBM trials [7,22]. Approximately 28% of patients had suboptimal KPS under 70. Over a quarter (26.2%) of patients had a Charlson comorbidity score above six, which generally reflects two comorbidities or an advanced comorbidity besides solid tumor in a person 71 years of age (median age of our cohort) or older. This comorbidity score was also the median for the entire cohort, irrespective of sex. Gross total resection (GTR) was achieved in 26 patients, and subtotal resection (STR) was performed on the remaining 35 patients. Over two-thirds (68.9%) of GBM tumors lacked methylation of the MGMT promoter. Approximately 44% of patients were government-insured and resided in a low-income zip code. Above one-third of patients (34.4%) were unmarried, widowed or without a domestic partner.

### 3.2. Univariate Analysis

**Figure 1:**
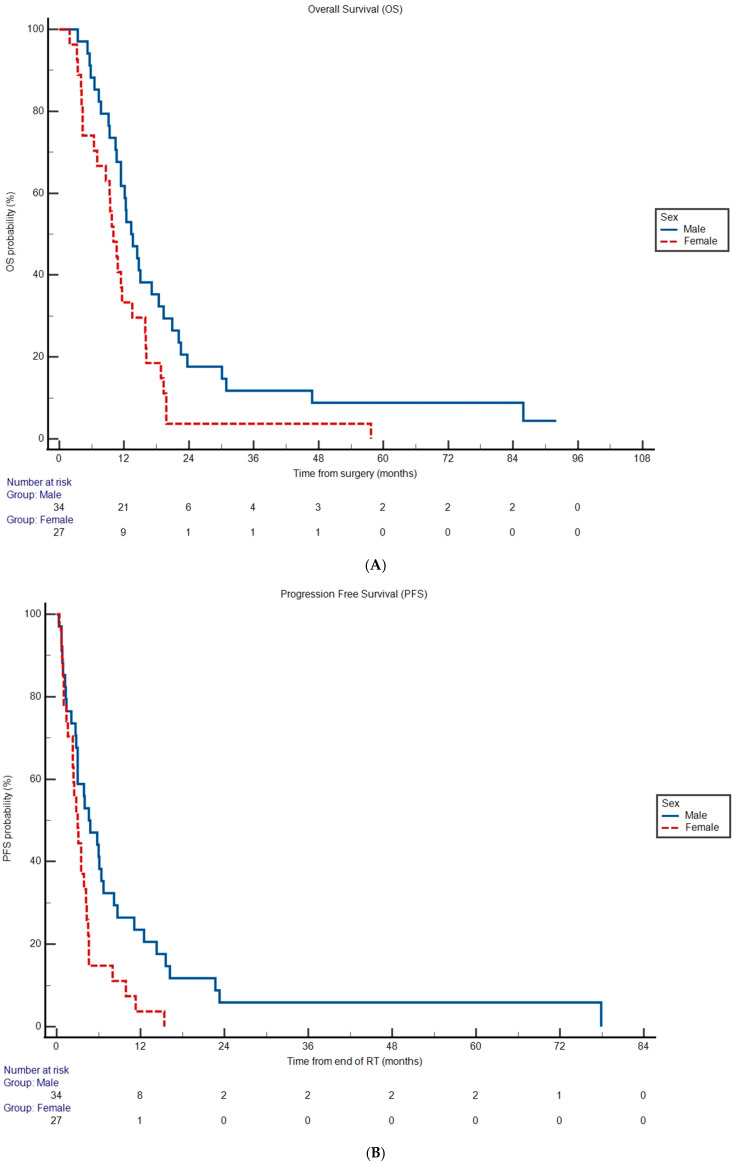
Overall survival (**A**) and progression-free survival (**B**) probabilities according to sex subgroup. Differences in Kaplan–Meier curves were statistically significant in both cases (*p* < 0.05). Tables under the graphs represent the remaining number of subgroup patients at risk for experiencing an event of interest at given timepoint.

Median OS was 11.6 months (95% CI 10.0 to 14.4) and median PFS was 3.50 months (95% CI 2.80 to 77.9) for the entire cohort. Lack of MGMT promoter methylation was associated with increased hazard rate of death (OS HR 2.11, 95% CI 1.24 to 3.59, *p* = 0.00570) and disease progression (PFS HR 2.50, 95% CI 1.45 to 4.32, *p* = 0.00100) compared to patients with MGMT promoter methylation (Table 2, Supplementary Figure S1A,B). Median OS was shorter in females (Figure 1A) at 10.0 months versus 13.3 months in males (HR 1.92, 95% CI 1.10 to 3.37, *p* = 0.0224; Table 2). Female patients also experienced an increased hazard rate of disease progression (HR 2.07, 95% CI 1.16 to 3.69, *p* = 0.0134; Table 2) compared to male patients with a median PFS of 3.00 versus 4.60 months, respectively (Figure 1B). The remainder of the covariates analyzed were not significantly associated with OS and PFS (*p* > 0.05; Table 2).

### 3.3. Multivariate Cox Proportional Hazard Analysis

In multivariable Cox regression analysis (Supplementary Table S1), MGMT promoter methylation status remained significantly associated with OS (HR 2.33, 95% CI 1.33 to 4.17, *p* = 0.00340) and PFS (HR 2.56, 95% CI 1.43 to 4.55, *p* = 0.00180). Similarly, sex remained significantly associated with OS (HR 2.02, 95% CI 1.18 to 3.45, *p* = 0.0108) and PFS (HR 1.91, 95% CI 1.11 to 3.30, *p* = 0.0191).

### 3.4. Chi-Squared Analysis for Covariate Association with Sex

Tumor variables such as MGMT methylation status, EOR and tumor multifocality did not differ significant between males and females (*p* > 0.05; Table 3). Similarly, clinical variables such as comorbidity score and functional status did not significantly differ between sex subgroups (*p* > 0.05; Table 3). Sex and marital status had a significant association, with more men being married and more women being single, divorced or widowed in our cohort (*p* = 0.000100; Table 3). The remainder of demographic variables, including income level and insurance status, did not differ between males and females (*p* > 0.05; Table 3).

## 4. Discussion

Emerging studies are reporting on the prognostic value of demographic factors like sex, income level and insurance status in GBM [10,11,12,16,17,23]. These studies converge with the accumulating neuro-oncology literature that demonstrates demographic-based differences in outcomes across a variety of brain tumors [24,25]. With regard to GBM specifically, the majority of these studies have been performed using the NCDB, which lacks outcomes data beyond OS and omits important RT-related details. However, an understanding of the impact of demographic factors on outcomes besides OS is important, as early-phase trials often rely on endpoints such as PFS to advance promising investigational therapies [26]. Equally important is accounting for specific RT details such as dose and technique, as the availability and completion of different radiation regimens (cvRT vs. hyRT) may be impacted differently by certain demographic variables (e.g., insurance status, geographic location). In this study, we evaluated the association of demographic factors with OS and PFS in a cohort of newly diagnosed GBM patients treated with hyRT-TMZ, an abbreviated chemoradiation schedule that has not been studied in the context of demographic prognostic variables.

In our cohort, females had significantly lower survival and shorter time to recurrence following hyRT-TMZ when compared to males. This difference in outcomes occurred despite comparable clinical (e.g., EOR, KPS) and pathological (e.g., MGMT promoter methylation) characteristics. Although sex-based differences in GBM outcomes have been previously reported in the literature, these reports have generally put forth the opposite association: namely, that female patients respond better to standard-of-care therapy than their male counterparts [16,17,27]. Indeed, findings like these are reflected in the 2021 NRG RTOG GBM nomogram and have culminated in the recent validation of a sex-specific version based on RTOG trials 0525 and 0825 [14,15]. However, it is important to note that these RTOG trials exclusively consisted of patients treated with cvRT-TMZ, and there have been no published reports to-date that focus on patients treated with hyRT-TMZ, a shorter-lived cohort in whom clinical discussions and decisions stand to benefit greatly from improved personalized prognostication.

Sexual dimorphism in GBM incidence, biology and outcomes has previously been described in the literature and is speculated to arise from hormonal, metabolic, genetic and immune differences between males and females [27,28]. Preclinical studies, for instance, demonstrate that estrogen increases the apoptosis of GBM cells and suppresses the oncogenic mitogen-activated protein kinase pathway in a sex-dependent manner [29]. Other immune studies in preclinical GBM models show a monocytic predominance in myeloid cells in male mice versus granulocytes in female mice, as well as differing cytokine profiles [30,31]. These studies in particular generally demonstrate a more pro-inflammatory tumor milieu in female versus male subjects. However, how biological factors such as estrogen, immune subsets or cytokines are modulated by cvRT versus hyRT, and to what extent this may impact GBM growth and recurrence, remains to be studied. At best, these preclinical studies provide a conjectural basis for differences in de novo GBM tumorigenesis between males and females, but do not offer or investigate a sex-dependent link between chemoradiation treatment and outcomes. Additional studies are therefore critically needed to elucidate how different chemoradiation regimens may benefit distinct sex subgroups, and how these may be applied to personalized treatment approaches in the clinic. Translational studies should consider capturing data in a sex-stratified manner from easily accessible tests (e.g., cytokine assay from peripheral blood, radiomic features from MRI) during the course of treatment to correlate with chemoradiotherapy outcomes prospectively.

Studying sex as a predictive variable for treatment response is highly relevant in GBM as new chemoradiation schedules are increasingly being tested in the clinic. Two U.S. institutions recently reported their experiences with dose-escalated hyRT, which delivers a biologically effective dose (BED) closer to cvRT and potentially confers survival gains over classical hyRT [32,33]. Sex-specific considerations in BED selection are particularly important in light of recent reports of female GBM patients harboring higher tumor volume and areas of necrosis on MRI compared to males [34]. If this is the case, it is possible that higher tumor and/or necrotic volume may necessitate a higher BED than that conferred by classical hyRT. While, in our study we did not observe sex differences in multifocality or subtotal resection rates—surrogates of disease burden—it is important to note that we did not directly quantitate tumor volume or necrosis. Future radiological and histological studies should, therefore, consider investigating the correlation of these metrics with sex and the possible impact on response to radiotherapy regimens with different BED levels. Potential immunophenotyping studies could also be performed in a BED-specific manner on tumor specimens collected post-radiotherapy at recurrence.

In our study, we did not find that insurance status or income based on zip code associated with treatment outcomes. This stands in contrast to prior studies demonstrating a correlation between government insurance/uninsured status as well as low-income to inferior outcomes in GBM [10,12,23]. It is possible that in our cohort of GBM patients receiving hyRT the disease was so aggressive that the impact of insurance status and income did not significantly influence treatment outcomes. Of note, we did observe that a higher proportion of females in our study were unmarried or without a domestic partner compared to males. Previous studies using the Surveillance, Epidemiology, and End Results (SEER) database demonstrate a protective effect of marriage in GBM outcomes, particularly in elderly males, and is congruent with observations in other types of cancer [10,35,36,37]. However, due to the sample size of our cohort we could not determine with high statistical confidence the degree to which this covariate influenced treatment response within sex subgroups. As mentioned, independently, marital status did not predict for OS or PFS. Additional studies are warranted to investigate the potential impact of marriage, companionship and family support in GBM patients undergoing hyRT.

While the results of this exploratory study are interesting and can be used to generate hypotheses, they must be considered in light of several limitations. First, the retrospective nature of this study limits our conclusions to associations between variables and clinical outcomes, and causality cannot be established. Secondly, the small sample size of our cohort, while comparable to other U.S. retrospective studies of elderly/frail GBM patients, further limits the ability to detect true differences between subgroups [38,39]. We recognize that large, prospective studies are needed in order to confirm any veritable relationship between sex and survival outcomes. Moreover, our study only included patients who completed hyRT in our analyses and, thus, we cannot account for any disparities that may exist in the referral and/or RT completion phases. While out of the scope of our original objectives, we did confirm that there were no major differences in median time to treatment completion between females (21 days, IQR 1.75) and males (22 days, IQR 1). Lastly, we were unable to ascertain differences in post-RT TMZ adherence, corticosteroid use, surveillance schedule and salvage therapies between patient subgroups due to limitations of available data points. Future studies should prospectively collect and assess the impact of these variables on outcomes.

## 5. Conclusions

Survival continues to be dismal for GBM patients treated with hypofractionated chemoRT. Additional research is needed to elucidate sex-based differences in outcomes based on the type of chemoradiotherapy schedule administered.

## Figures and Tables

**Table 1:** Composite demographics of study cohort. KPS = Karnofsky Performance Status. MGMT = methylguanine-DNA methyltransferase.

Clinical and Demographic Characteristics	Number (%)
	61 (100%)
Age [median 71.0 years Interquartile Range 9.5 years]	
<65 years	9 (14.8%)
≥65 years	52 (85.2%)
Sex	
Male	34 (55.7%)
Female	27 (44.3%)
Extent of Resection	
Subtotal Resection	35 (57.4%)
Gross Total Resection	26 (42.6%)
Multifocal	
No	55 (90.2%)
Yes	6 (9.8%)
MGMT Status	
Unmethylated	42 (68.9%)
Methylated	19 (31.1%)
KPS	
<70	17 (27.9%)
≥70	44 (72.1%)
Comorbidity Score	
≤6	45 (73.8%)
>6	16 (26.2%)
Insurance	
Government	27 (44.3%)
Private	34 (55.7%)
Zip code Income	
Low	27 (44.3%)
Middle/High	34 (55.7%)
Marital Status	
Single/Divorced/Widowed	21 (34.4%)
Married/Domestic Partner	40 (65.6%)
Race	
White	42 (68.9%)
Non-White	19 (31.1%)

**Table 2:** Univariate analysis assessing association of covariates with overall survival (OS) and progression-free survival (PFS). Hazard ratio = HR. CI = confidence interval. KPS = Karnofsky Performance Status. MGMT = methylguanine-DNA methyltransferase.

Covariates	OS		PFS	
	HR (95% CI)	*p*	HR (95% CI)	*p*
Age				
continuous	1.00 (0.974–1.03)	0.999	0.999 (0.973–1.03)	0.957
Age (dichotomous)				
<65 years	reference	0.805	reference	0.545
≥65 years	1.10 (0.526–2.29)		1.24 (0.616–2.50)	
Sex				
Male	reference	0.0224	reference	0.0134
Female	1.92 (1.10–3.37)		2.07 (1.16–3.69)	
Extent of Resection				
Subtotal Resection	reference	0.338	reference	0.185
Gross Total Resection	0.776 (0.462–1.30)		0.704 (0.418–1.18)	
Multifocal				
No	reference	0.657	reference	0.476
Yes	1.23 (0.490–3.10)		1.42 (0.539–3.76)	
MGMT Status				
Methylated	reference	0.00570	reference	0.00100
Unmethylated	2.11 (1.24–3.59)		2.50 (1.45–4.32)	
KPS				
<70	reference	0.244	reference	0.836
≥70	0.685 (0.362–1.29)		0.940 (0.521–1.69)	
Comorbidity Score				
≤6	reference	0.474	reference	0.701
>6	1.25 (0.677–2.31)		1.12 (0.619–2.04)	
Insurance				
Private	reference	0.835	reference	0.385
Government	1.06 (0.624–1.79)		1.27 (0.743–2.16)	
Zip Code Income				
Middle/High	reference	0.863	reference	0.827
Low	0.954 (0.558–1.63)		1.06 (0.626–1.80)	
Marital Status				
Married/Domestic Partner	reference	0.705	reference	0.719
Single/Divorced/Widowed	1.11 (0.641–1.93)		1.11 (0.635–1.93)	
Race				
White	reference	0.931	reference	0.530
Non-White	1.03 (0.588–1.70)		0.839 (0.485–1.45)	

**Table 3:** Assessment of differences in clinical, tumor and demographic factors between males and females by Chi-square analysis. pX^2^ = *p* value for chi-square analysis. KPS = Karnofsky Performance Status. MGMT = methylguanine-DNA methyltransferase.

Covariates		Sex			Covariates		Sex		
	Total	Male	Female			Total	Male	Female	
	61	34 (55.7%)	27 (44.3%)			61	34 (55.7%)	27 (44.3%)	
MGMT Status				*p*X^2^	Comorbidity Score				*p*X^2^
Unmethylated	42 (68.9%)	23 (67.6%)	19 (70.4%)	0.821	≤6	45 (73.8%)	25 (73.5%)	20 (74.1%)	0.962
Methylated	19 (31.1%)	11 (32.4%)	8 (29.6%)		>6	16 (26.2%)	9 (26.5%)	7 (25.9%)	
Extent of Resection					Insurance Status				
Subtotal Resection	35 (57.4%)	20 (58.8%)	15 (55.6%)	0.799	Government	27 (44.3%)	12 (35.3%)	15 (55.6%)	0.117
Gross Total Resection	26 (42.6%)	14 (41.2%)	12 (44.4%)		Private	34 (55.7%)	22 (64.7%)	12 (44.4%)	
Multifocal					ZipCodeIncome				
No	55 (90.2%)	29 (85.3%)	26 (96.3%)	0.155	Low	27 (44.3%)	17 (50.0%)	10 (37.0%)	0.315
Yes	6 (9.8%)	5 (14.7%)	1 (3.7%)		Middle/High	34 (55.7%)	17 (50.0%)	17 (63.0%)	
Age					MaritalStatus				
<65 years	9 (14.8%)	7 (20.6%)	2 (7.4%)	0.152	Single/Divorced/Widowed	21 (34.4%)	4 (11.8%)	17 (63.0%)	0.000100
≥65 years	52 (85.2%)	27 (79.4%)	25 (92.6%)		Married/DomesticPartner	40 (65.6%)	30 (88.2%)	10 (37.0%)	
KPS					Race				
<70	17 (27.9%)	10 (29.4%)	7 (25.9%)	0.765	White	42 (68.9%)	26 (76.5%)	16 (59.3%)	0.153
≥70	44 (72.1%)	24 (70.6%)	20 (74.1%)		Non-White	19 (31.1%)	8 (23.5%)	11 (40.7%)	

## Data Availability

The data used and analyzed during the current study are available from the corresponding authors upon reasonable request.

## Supplementary Materials

The following supporting information can be downloaded at: https://www.mdpi.com/article/10.3390/cancers17213486/s1, Figure S1: Kaplan-Meier (A) overall survival (OS) and (B) progression-free survival (PFS) estimates for patient cohort stratified by methylguanine-DNA methyltransferase (MGMT) promoter methylation status. Differences in Kaplan-Meier curves were statistically significant in both cases (*p* < 0.05); Table S1: Multivariable Cox proportional hazards analysis for overall survival (OS) and progression-free survival (PFS). Hazard ratios (HR) with 95% confidence intervals (CI). MGMT = methylguanine methylation.

## Abbreviations

The following abbreviations are used in this manuscript:

GBM	glioblastoma
hyRT	hypofractionated radiotherapy
cvRT	conventionally fractionated radiotherapy
cGy	centi-Gray
TMZ	temozolomide
OS	overall survival
PFS	progression-free survival
EOR	extent of resection
MGMT	methylguanine-DNA methyltransferase
KPS	Karnofsky Performance Scale
NCDB	National Cancer Database
RTOG	Radiation Therapy Oncology Group
SEER	Surveillance, Epidemiology, and End Results
MRI	Magnetic Resonance Imaging
BED	Biologically Effective Dose
