# Supplementary material for: Sex-Based Differences in Outcomes for Glioblastoma Patients Treated with Hypofractionated Chemoradiotherapy
Source: Cancers (Basel). 2025 Oct 30;17(21):3486. doi: 10.3390/cancers17213486 (PMC12607755; doi:10.3390/cancers17213486)
Supplement: Supplementary file 1 [file cancers-17-03486-s001.zip › cancers-3929504-supplementary.pdf]

## Supplementary Material

**Supplementary Table S1.** Multivariable Cox proportional hazards analysis for overall survival (OS) and progression-free survival (PFS). Hazard ratios (HR) with 95% confidence intervals (CI). MGMT = methylguanine methylation.

| Covariates | OS model | | PFS model | |
|---|---|---|---|---|
| | HR (95% CI) | *p* | HR (95% CI) | *p* |
| **MGMT Status** | | | | |
| **Methylated** | reference | | reference | |
| **Unmethylated** | 2.33 (1.33-4.17) | 0.00340 | 2.56 (1.43-4.55) | 0.00180 |
| **Sex** | | | | |
| **Male** | reference | | reference | |
| **Female** | 2.02 (1.18-3.45) | 0.0108 | 1.91 (1.11-3.30) | 0.0191 |
| **Overall Model** | | 0.000900 | | 0.000300 |

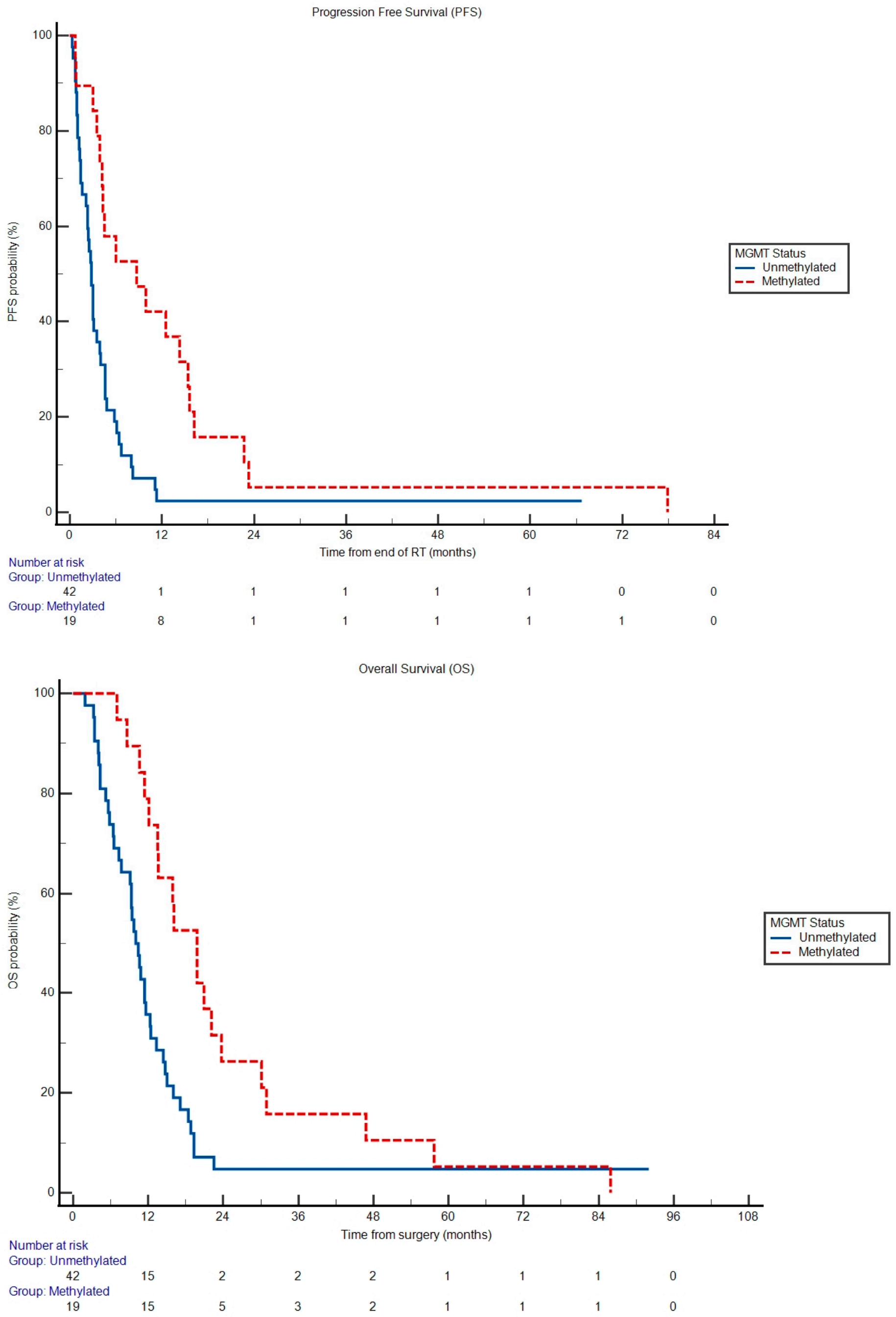


**Supplementary Figure S1.** Kaplan-Meier (A) overall survival (OS) and (B) progression-free survival (PFS) estimates for patient cohort stratified by methylguanine-DNA methyltransferase (MGMT) promoter methylation status. Differences in Kaplan-Meier curves were statistically significant in both cases ($p<0.05$). Tables under the graphs represent the remaining number of subgroup patients at risk for experiencing event of interest at given timepoint.
